# A Typology of Strategies Women Use to Navigate Alcohol Abstinence During Pregnancy

**DOI:** 10.1007/s10995-026-04281-0

**Published:** 2026-05-26

**Authors:** Simone Pettigrew, Bella Sträuli, Leon Booth, Keira Bury, James Stevens-Cutler, Annet Hoek

**Affiliations:** 1 University of New South Wales, Sydney, Australia; 2 Griffith University, Brisbane, Australia; 3 The George Institute for Global Health, University of New South Wales, Sydney, Australia; 4 Western Australia Mental Health Commission, Perth, Australia

**Keywords:** Alcohol, Pregnancy, Prevention, Communications

## Abstract

**Introduction:**

Current guidelines recommend alcohol abstinence during pregnancy and while trying to conceive to prevent avoidable harms. However, social norms favoring alcohol use can make abstinence difficult, especially while trying to conceal a pregnancy. The aim of this study was to identify feasible alcohol avoidance strategies and categorize them into a typology that can be used to communicate about this topic with women of child-bearing age.

**Methods:**

Online focus groups (*n* = 37) and individual interviews (*n* = 6) were conducted with 43 Australian women aged 18–44 years who were pregnant or had recently been pregnant at the time of data collection and who had consumed alcohol at least weekly before conception. Projective techniques were used that involved presenting participants with scenarios of fictional women in situations that may arise when trying to avoid alcohol during pregnancy.

**Results:**

Findings indicate that those who are pregnant or trying to conceive may benefit from ready access to information about alcohol avoidance strategies that have been found effective by the women who went before them. Identified strategies were classified as Avoid/constrain the drinking context, Substitute drinks, Use an accomplice, Simulate drinking, Fib, Educate, Refuse, and Disclose.

**Discussion:**

These strategies could form the basis of health practitioner advice and user-friendly resources that are made available to women of childbearing age and pregnant women to increase their ability to avoid alcohol while trying to conceive and during pregnancy.

## Significance

What is already known on this subject?

What this study adds?

Alcohol abstinence during pregnancy and while trying to conceive is recommended, but avoiding alcohol can be challenging in social contexts where drinking is normalized. Only a limited range of strategies that women can use to achieve abstinence at this time of their lives has been identified in previous research.

This qualitative study developed a typology of eight alcohol-avoidance strategy categories used by Australian women to abstain from alcohol during pregnancy and pre-conception. The findings highlight the value of providing women with practical, user-friendly guidance and examples of feasible strategies that may support alcohol abstinence during pregnancy.

## Introduction

During pregnancy, alcohol passes through the placenta to produce comparable blood alcohol levels in mother and fetus (Burd et al., 2012). Permanent adverse outcomes resulting from alcohol exposure can include impaired development, fetal alcohol spectrum disorder, and stillbirth (DeJong et al., 2019). World Health Organization guidance is that there is no safe level of alcohol use in pregnancy, and that both pregnant women and those trying to conceive should avoid alcohol (World Health Organization, 2023).

Globally, around one in ten women use alcohol during pregnancy (Popova et al., 2017). This occurs most often in the early stages before pregnancy is confirmed (Ishitsuka et al., 2020; Tsang et al., 2022). In Australia, abstinence recommendations for pregnancy have been in place since 2009 (National Health and Medical Research Council, 2009). However, although declining over time, the proportion of Australian women consuming alcohol during pregnancy remains high at around 28% (Australian Institute of Health and Welfare, 2024).

Avoiding alcohol during the pre-conception phase and before pregnancy status is confirmed or disclosed can be challenging in cultures where alcohol use is normalized (Pistone et al., 2024), making it difficult for women to discretely abstain (Gibson et al., 2020; Gouilhers et al., 2019; Meurk et al., 2014). Around 20% of pregnancies end in miscarriage, most often in the first trimester (Avalos et al., 2012), and many women choose to conceal pregnancy until after this period (Hutson, 2025). In such situations, women may need to identify and use strategies to avoid alcohol while preventing speculation about their pregnancy status.

While there is a substantial body of research on protective behavioral strategies used by various other population groups (Dekker et al., 2020; DiGuiseppi et al., 2025), there is very limited evidence on the types of strategies women can and do use to avoid alcohol during pregnancy. The few studies that have specifically addressed this issue have concluded that (i) providing examples of effective strategies is likely to be appreciated (France et al., 2013, 2014), (ii) partners can be important sources of support (Gouilhers et al., 2019), (iii) awareness of strategies to avoid alcohol during pregnancy is associated with more favorable perceptions of abstinence messages (Pettigrew et al., 2023), and (iv) assisting women to implement effective alcohol avoidance strategies is an important element of a comprehensive approach to supporting abstinence during pregnancy (Deutsch et al., 2021). To provide additional insights, the aims of this study were to (i) explore women’s experiences with alcohol-avoidance strategies and (ii) develop a typology of strategies women have successfully used to abstain from alcohol when pregnant or planning a pregnancy to inform efforts to support women wanting to avoid alcohol during this period.

## Methods

A qualitative research approach was used involving online focus groups and interviews featuring projective techniques in the form of simulated scenarios to explore women’s perceptions and experiences relating to pregnancy and alcohol avoidance. The combination of focus groups and interviews catered for the availabilities and preferences of participants. Ethics approval was obtained for the project from a university Human Research Ethics Committee. All participants provided written informed consent and received AU$100 remuneration.

### Sample

Forty-three women participated across six focus groups (*n* = 37) and six individual interviews (*n* = 6). Two social research data collection agencies (Thinkfield and Pureprofile) with ISO-accreditation (i.e., independently audited and verified to meet specific international standards) were commissioned to undertake participant recruitment. Eligible individuals were women aged 18–44 years who were currently pregnant or had given birth in the last three years, resided in Western Australia, and consumed alcohol at least weekly prior to pregnancy. Sample variation was sought across two age groups (18–30, 31–44 years), two income categories (lower or higher than the national household median annual income of AU$92,000 (Australian Bureau of Statistics, 2023), metropolitan versus non-metropolitan location, and level of alcohol use prior to pregnancy (complied with versus exceeded the low-risk guideline of ≤ 10 drinks per week and ≤ 4 drinks on any day (National Health and Medical Research Council, 2020) (see Table 1).

### Data Collection

**Fig. 1:**
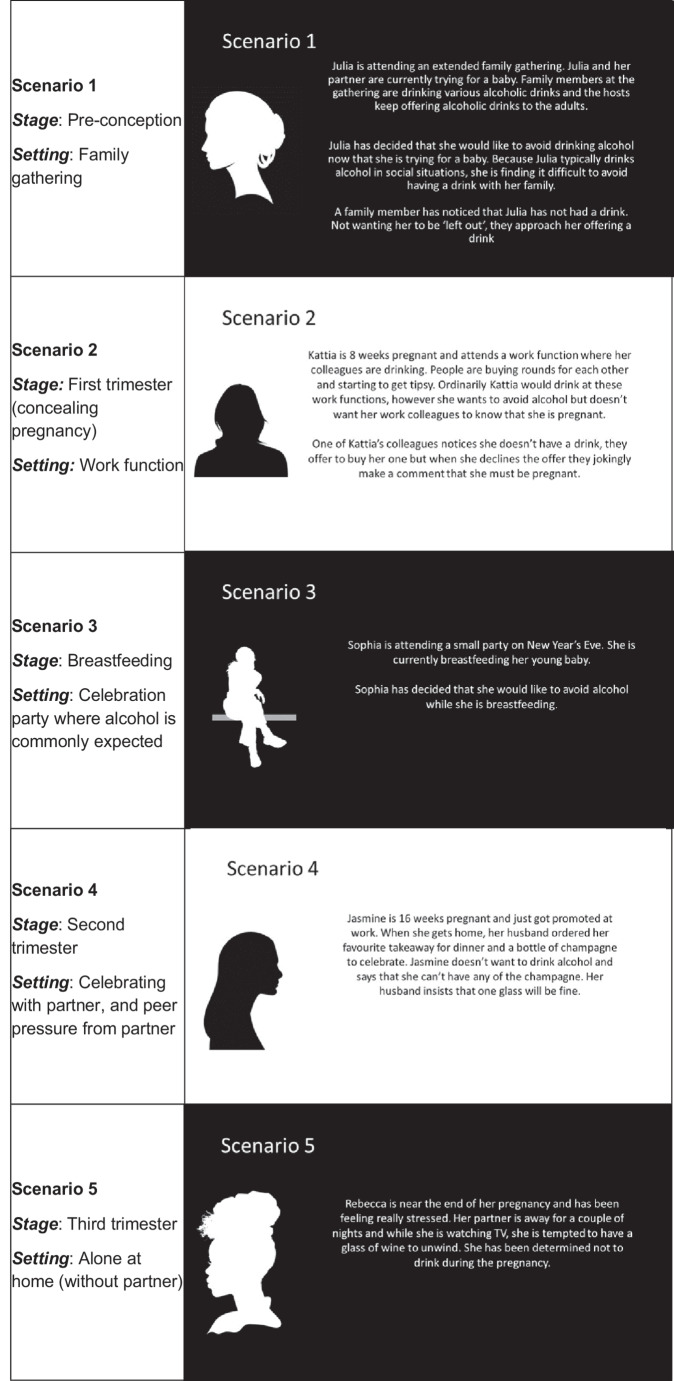
Stimuli shown to participants

Data collection occurred July-August 2024. A highly experienced qualitative researcher moderated the focus groups (author SP), and the individual interviews were conducted by the same researcher and a trainee researcher acting under supervision (author BS). The discussion guide included projective stimuli to prompt conversations about alcohol-related situations across the pregnancy journey. Projective techniques involve research participants responding to ambiguous stimuli and projecting their thoughts and feelings into their responses to access subconscious motivations and attitudes (Pettigrew & Roberts, 2011). In the present study, the projective stimuli comprised text descriptions of five scenarios applying to different stages of pregnancy, accompanied by silhouette images of females (the stimuli are shown in Fig. 1). The scenarios were developed by the author team and covered the five stages of pre-conception, first trimester, second trimester, third trimester, and breastfeeding, and comprised short accounts of a particular issue experienced by a notional person. For example, the second-trimester scenario stated: “Jasmine is 16 weeks pregnant and just got promoted at work. When she gets home, her husband ordered her favorite takeaway for dinner and a bottle of champagne to celebrate. Jasmine doesn’t want to drink alcohol and says that she can’t have any of the champagne. Her husband insists that one glass will be fine.”.

General prompts were used (e.g., “What do we think about the situation described in the image?”) to enable participants to interpret the depicted scenarios in any way they chose. When responding, participants were not required to disclose their own behaviors unless they wished to, and instead they could use the scenarios to anchor their discussions if preferred. No new concepts were emerging by the end of data collection, indicating conceptual saturation had been achieved. On average, the focus groups were 88 min duration and the interviews 49 min.

### Data Analysis

Session recordings were transcribed verbatim and the transcripts imported into NVivo software for coding and analysis. Line-by-line coding, key word searches, and matrix searches were used by the lead author (SP) to assign transcript content to topic codes. Codes applied during the data coding process were a combination of deductive codes based on the topics included in the interview guide and inductive codes representing concepts raised by the participants. An emergent coding hierarchy was created based on the deductive and inductive codes to provide a framework for analysis. The use of a single coder was appropriate due to the coding framework evolving across the course of the study (Smith & McGannon, 2018). A thematic approach to data interpretation involved application of the constant comparative method to identify similarities and differences between participants’ accounts (Glaser & Strauss, 1967).

## Results

Participants readily shared their personal experiences with alcohol during pregnancy while also discussing the options available to the fictional women depicted in the scenarios. Example quotes are provided below using the following descriptor key: age (in years), income level (lower, higher), location (metro, non-metro), compliance with low-risk drinking guideline prior to pregnancy (complied, exceeded), and interview type (FG (focus group), IDI (individual depth interview)).

The strategies the study participants reported using to avoid alcohol almost always pertained to the first trimester due to the desire to conceal pregnancy during this time. Some of the participants reported learning about effective strategies from friends who had gone before them and shared their experiences, while others had tried to come up with ideas on the spot and felt unprepared. Many of the focus group participants appeared to enjoy hearing about each others’ strategies, as indicated by smiles and nods, and reported to the group that they planned to use the new ideas they heard.

I’m not quick on my feet with coming up with an answer, so I think that’s why I’m going to use some of these ones that people are coming up with in the future, because you obviously need to have a little bank of replies ready to go really quick back to people. If you’re not quick on your feet, you panic (37, higher income, metro, exceeded, FG).

There was general agreement that it would be useful for women to be able to easily access information about effective strategies to assist them avoid alcohol during their first trimester of pregnancy. In some instances, participants felt such a resource would be useful for themselves, while others saw it as being more beneficial for others (e.g., anxious or first-time mothers).

I think it’s a good idea to have a website that you could go to, to be able to see that sort of stuff, different strategies that you could use (36, lower income, regional, exceeded, FG).

If you were unsure, if you were more likely inclined to have no idea and it makes you really anxious about it, then I think having something that’s going to help prepare for that is never a bad thing (40, higher income, metro, complied, FG).

As shown in Table 2, the strategies discussed by the study participants were classified into eight behavioral categories. Five involved ‘misdirects’ to disguise non-drinking (Avoid/constrain the drinking context, Substitute drinks, Use an accomplice, Simulate drinking, and Fib) and three involved adopting a more forthright approach when interacting with others (Educate, Refuse, and Disclose).

### Avoid/Constrain the Drinking Context

References to using strategies that involve entirely or partially avoiding drinking events were often made in response to the scenario in Fig. 1 describing the woman in her first trimester attempting to conceal her pregnancy at a work drinks event. This situation resonated with many of the study participants, with descriptions provided about how they had pre-emptively anticipated the need to either opt out altogether or leave early. This approach appeared to be more common among the older participants and those who had complied with the low-risk drinking guideline prior to pregnancy.

At a work event, I’ve just said, “I’ve got a family dinner on, I have to be there at 6”. So I’d leave at 5 and just end the night early. Once again, unfortunately, have that script ready to go, just to try to avoid the situation altogether (34, higher income, metro, complied, FG).

A common reason the participants used to explain leaving early during their first trimester was needing to drive. This appeared to be widely accepted as a legitimate reason for abstaining from alcohol during events and as such was an effective way to fend off expectations to drink.

Say that you’re the designated driver today and you step out of drinking because you say you’re driving. Usually people are pretty understanding about that (35, lower income, metro, exceeded, IDI).

### Substitute Drinks

There was a general understanding that people offering alcohol were trying to be hospitable, and therefore could be alleviated of this concern by the pregnant person always having a substitute drink in hand. Some participants reported thinking ahead and bringing their own drinks to ensure they always had a supply of non-alcoholic beverages on hand to choose from at an event to prevent others from attempting to provide them with alcohol.

If you have a drink in your hand, people are not going to question whether it’s alcoholic or not, as long as you have a drink in your hand (29, lower income, metro, exceeded, IDI).

### Use an Accomplice

Abstinence accomplices were partners or friends who were aware of the individual’s pregnancy status and assisted them in avoiding alcohol before the pregnancy was formally announced. Most often the accomplice was the pregnant woman’s partner, but sometimes female friends took on this role. The actions taken by accomplices included fetching non-alcoholic drinks disguised as alcoholic drinks and assuring well-meaning hosts that they were looking after their partner’s/friend’s drinks.

We might bring a gin and tonic or something that is a clear drink, and send my husband off to go make our drinks, but obviously make mine a virgin one (34, higher income, metro, complied, FG).

I just had one good friend who knew I was pregnant, and they would go and buy my drinks for me. It would just be lemonade, but everyone would think I’m drinking vodka lemonade (22, lower income, regional, complied, IDI).

A further strategy was for the accomplice to consume the pregnant woman’s drink when no-one was looking. However, this does not appear to be an advisable strategy due to the potential for the accomplice’s alcohol use to increase relative to their normal intake due to ‘drinking for two’.

I might surreptitiously pass the drink to hubby after accepting it. Like, “Quick, down this” (38, lower income, regional, exceeded, FG).

In some instances, the participants mentioned solidarity in numbers by abstaining with someone else. Examples included the partner also electing to abstain so the pregnant person did not appear different and making the most of a friend or colleague being pregnant at the same time.

One of my colleagues at work was also pregnant, but she was a lot further along than me. So when she ordered a particular mocktail off a menu, I just piggybacked off that and said, “Oh, that sounds really good, I’ll have one of those too” (33, higher income, metro, complied, FG).

### Simulate drinking

Pretending to drink an alcoholic beverage but not actually consuming it was a very common strategy used in the first trimester, especially among the older participants. This strategy focused on causing others to believe that alcohol was being consumed without involvement of others to assist in the deception. This was done in three main ways. The first was drinking from glassware typically only used for alcoholic beverages and/or choosing products that looked like they could be alcohol (e.g., soft drinks that could be spirits with mixers).

You have to pretend that you’re drinking. So, I’ve done in the past soda water in a champagne glass so people think that you’re partaking in the drinking. Or I’ve ordered a mocktail instead of a cocktail so that at work functions they wouldn’t know that I was pregnant (34, lower income, metro, exceeded, FG).

The second form of simulation involved consuming a zero-alcohol product while leading others to think it was a normal alcoholic beverage. Participants did not appear to be aware that zero-alcohol products can contain small amounts of alcohol (Bowdring et al., 2024).

I would have a non-alcoholic red wine that I’d pour into my glass and then pour the regular wine into other people’s glasses. So, by the time we sat down, no one could tell the difference (34, higher income, metro, complied, FG).

The third simulation strategy was accepting the alcoholic drink but discarding it when no-one was looking. This could involve tipping the drink out or just setting the drink down and walking away.

You take your drink with you to the toilet and you tip it down the sink. You fill it up with water, and then you’re back out there and you’re taking sips, and it looks like you’re drinking out of the can (22, lower income, regional, complied, IDI).

I would’ve just took it and kind of held it in my hand all night or put it down somewhere (31, higher income, metro, exceeded, FG).

### Telling Fibs

Many participants reported making up phantom reasons for not drinking to avoid alcohol in social situations. This approach seemed to be the least effortful option and could be implemented on one’s own (i.e., without an accomplice) and with little prior planning. While some of the participants referred to having pre-prepared scripts, others made up fibs on the spot. Common examples included saying they were (i) experiencing health problems, such as taking medication, being generally unwell, or feeling hungover; (ii) on a health kick/diet; (iii) committed to specific activities the next morning, such as being on child-minding duty or playing sport/working out; and (iv) on a ‘dry month’, such as Dry July. Participants routinely mentioned using multiple fibs throughout their pregnancies, demonstrating that having a repertoire was useful to cater for different situations.

### Educate

Partners and older female relatives were mentioned most often as needing to be educated about the potential effects of alcohol on the unborn child. While almost all of the study participants reported strong partner support for abstaining during pregnancy, the scenario describing a promotion celebration (Scenario 4, see Fig. 1) yielded discussions about conversations with partners to either proactively ensure they were educated about alcohol risks or, in a small number of cases, to respond to their partners’ efforts to encourage them to drink. These instances were typically raised by participants who reported exceeding the low-risk guideline prior to pregnancy.

I used the scare tactic and sent him all the bad things that can happen and all the foods and things you can’t really eat. I think he would be supportive regardless, but just having that peace of mind that he’d understand a lot more just helped (19, higher income, metro, exceeded, FG).

When discussing unwelcome pressure to drink from the older women in their lives, the knowledge held by these individuals was uniformly described as outdated. Participants reported being very willing to insist that the evidence had moved on.

Educate them, show them the research that’s been out since they were pregnant and how things have changed. Because sometimes they don’t actually know, they just think they know best (31, higher income, metro, exceeded, FG).

### Refuse

A few study participants were adamant that no explanation is required for refusing alcohol. They felt that anyone, pregnant or not, should be able to decline alcohol without providing an explanation. For these participants, any continued pressure once an offer of a drink was refused was offensive and should be dealt with decisively.

The more someone tries to get me to do something, the more I’m like, “No thanks”. And it’s like a hard no … Just say, “I’ve said no”. That’s what that means, it’s a whole sentence. No is a full sentence (29, higher income, regional, complied, IDI).

### Disclose

This strategy was most often reported by those who were older and on higher incomes. In some instances, the participants mentioned specific people in whom they would confide because they were special friends/family members or because they were most likely to be the ones with whom they interact in alcohol-infused situations.

I told my neighbors, at this stage, I said, well, not that we’re trying, but we’re not avoiding, so I just decided to stop drinking for a little while and then she was like, “Oh, I get it”. Then she stopped asking anything (37, higher income, metro, complied, FG).

At other times the participants reported telling everyone they were pregnant, which served to immediately shut down offers of alcohol or pressure to drink, thereby avoiding the complications associated with attempting to conceal the pregnancy.

There’s no need to hide the first trimester of your pregnancy. As soon as I know, I tell people and I stop drinking, and it’s clear why I’ve stopped drinking (22, lower income, regional, complied, IDI).

## Discussion

The study participants were supportive of sharing a repertoire of potentially feasible alcohol avoidance strategy options to those wishing to discretely abstain. The typology developed in the present study appears to be among the most comprehensive categorizations of the types of strategies that can be used to avoid alcohol during pregnancy, and as such could be a useful resource. Previous research has identified some of the individual strategies; for example, in an earlier Australian study women reported telling fibs (Jones & Telenta, 2012), and in a Swiss study women reported switching drinks with their partners to avoid raising suspicion (Gouilhers et al., 2019). By categorizing the large number of strategies into eight major categories, the typology may provide useful structure for presenting alcohol avoidance strategy information to those who could benefit.

While the full typology is useful to understand behaviors, it is recommended that two components are removed prior to broad dissemination: (i) asking an accomplice to consume the pregnant person’s drink (this could result in the partner consuming excessive quantities of alcohol) and (ii) encouraging the use of zero-alcohol products (which can have a small quantity of alcohol present (Bowdring et al., 2024). These strategies may have potential adverse outcomes and as such may not be suitable for endorsement.

The very limited prior work in this area necessitated an exploratory approach to data collection that involved a modest sample, albeit larger than for some previous research examining the issue of avoiding alcohol during pregnancy (Gibson et al., 2020; Jones & Telenta, 2012), and there was the potential for social desirability bias to have impacted the findings. The study results cannot be considered generalizable and additional research is required to assess the extent to which the identified alcohol avoidance strategies are relevant to a broader sample of Australian women and those in other countries with different alcohol norms. Other potential areas of further research include investigating specific messages to disseminate the alcohol avoidance strategies identified in this study.

**Table 1:** Sample composition (*n* = 43)

Characteristic	*n*	%
Age		
18–30 years	16	37
31–44 years	27	63
Household income^†^		
Lower	19	44
Higher	24	56
Location		
Metropolitan area	36	84
Non-metropolitan area	7	16
Pre-pregnancy alcohol use^‡^		
Within low-risk guideline	17	40
Exceed low-risk guideline	26	60
Pregnancy status		
Currently pregnant	6	14
Recently pregnant	37	86

^†^Below and above household income median of AU$92,000 p.a. (Australian Bureau of Statistics, 2023).

^‡^As per National Health and Medical Research Council’s low-risk guideline of no more than ten drinks per week and no more than four drinks on a single drinking occasion (National Health and Medical Research Council, 2020).

**Table 2:** Strategies used to avoid alcohol use in the first trimester

Misdirect strategies					Direct strategies		
Avoid/constrain the drinking context	Substitute	Use an accomplice	Simulate drinking	Fib	Educate	Refuse	Disclose pregnancy status
Stay away	Choose other non-alcoholic beverages	Get drinks	Mineral water in champagne glass	On medication/unwell/hangover	Partner	Say no without explanation	To close friends/family
Leave early	Bring own beverage	Consume the pregnant person’s drink†	Zero-alcohol products^†^	Health kick/diet	Family		To everyone
Drive		Abstain together	Hold and discard	Child- minding duty			
				Sport			

^†^Exclude from education resources due to potential adverse consequences

In conclusion, those who are pregnant or trying to conceive may appreciate ready access to information about alcohol avoidance strategies that have been found effective by the women who went before them. The strategies identified in this study could form the basis of health practitioner advice and user-friendly resources that are made available to women to give them access to a bank of strategies that may assist them to avoid alcohol during pregnancy and while trying to conceive.

## Data Availability

Ethics approval for this study does not permit data sharing.
